# Supplementary material for: Brain Activity Reveals Multiple Motor-Learning Mechanisms in a Real-World Task
Source: Front Hum Neurosci. 2020 Sep 2;14:354. doi: 10.3389/fnhum.2020.00354 (PMC7492608; doi:10.3389/fnhum.2020.00354)
Supplement: Supplementary file 1 [file Data_Sheet_1.PDF]

Supplementary Material

Brain activity reveals multiple motor-learning mechanisms in a real-world task

Shlomi Haar & A. Aldo Faisal

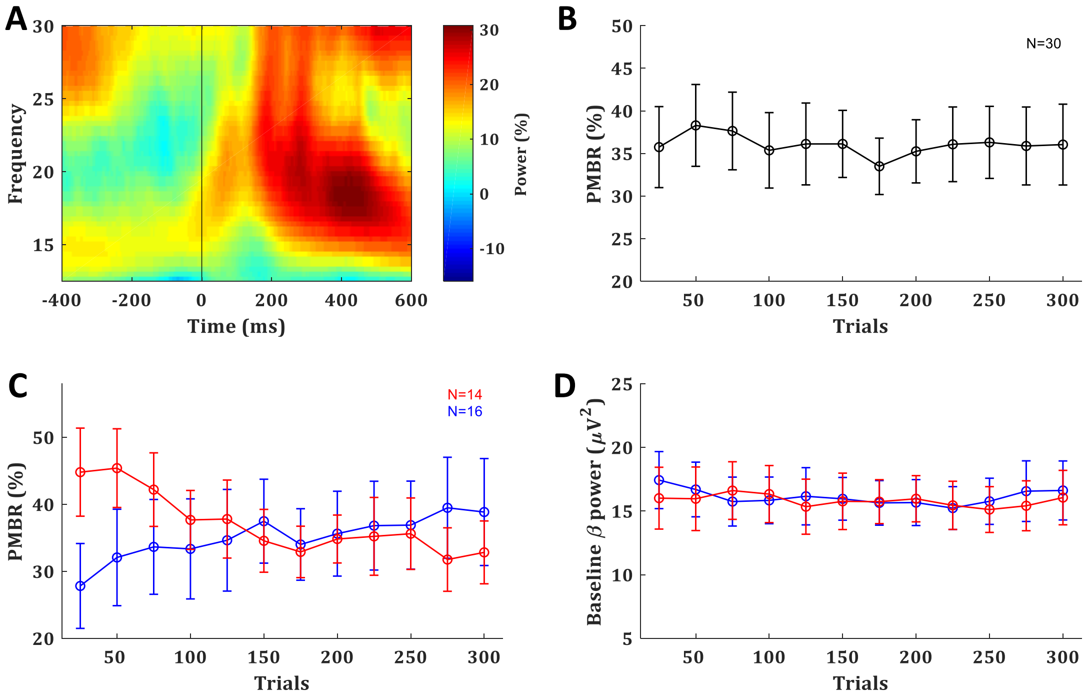

Right Motor Cortex Post-movement beta rebound. (**A**) Time-frequency map of a typical subject aligned to movement offset (ball movement onset), obtained by averaging the normalized power over electrode C4. (**B**) PMBR over blocks (of 25 trials), averaged across all subjects, error bars represent SEM. (**C,D**) PMBR (C) and Baseline beta power (D) of the *PMBR Increasers* (blue) and *PMBR Decreasers* (red) over blocks, averaged across all subjects in each groups, error bars represent SEM.
